# First Global Report of Plasmid-Mediated *mcr-1* and Extended-Spectrum Beta-Lactamase-Producing *Escherichia coli* from Sheep in Portugal

**DOI:** 10.3390/antibiotics10111403

**Published:** 2021-11-16

**Authors:** Josman Dantas Palmeira, Marisa Haenni, Jean-Yves Madec, Helena Maria Neto Ferreira

**Affiliations:** 1 Microbiology, Biological Sciences Department, Faculty of Pharmacy, University of Porto, 4050-313 Porto, Portugal; hferr@ff.up.pt; 2 UCIBIO—Applied Molecular Biosciences Unit, REQUIMTE—University of Porto, 4050-313 Porto, Portugal; 3 Departamento de Biologia & CESAM, Campus de Santiago, Universidade de Aveiro, 3810-193 Aveiro, Portugal; 4 PICTIS—International Platform for Science, Technology and Innovation in Health, Universidade de Aveiro (Portugal) & FIOCRUZ, Rio de Janeiro 21040-360, Brazil; 5 Unité Antibiorésistance et Virulence Bactériennes, Anses Laboratoire de Lyon, Université de Lyon, 69007 Lyon, France; Marisa.HAENNI@anses.fr (M.H.); Jean-Yves.MADEC@anses.fr (J.-Y.M.)

**Keywords:** *mcr-1*, extended-spectrum beta-lactamase, *E. coli*, sheep, Portugal

## Abstract

Resistances to extended-spectrum cephalosporins (ESC) and colistin are One Health issues since genes encoding these resistances can be transmitted between all sectors of the One Health concept, i.e., human, animal, and the environment. Among food-producing animals, sheep farming has long been overlooked. To fill in this knowledge gap, we looked for ESC- and colistin resistance in 21 faecal samples collected from sheep in one farm in the south of Portugal. ESC-resistant isolates were selected on MacConkey agar plates supplemented with cefotaxime. Susceptibility testing was performed by the disk-diffusion method according to CLSI, while colistin MIC was determined by broth microdilution. ESC- and colistin-resistance genes were identified by PCR, and the clonality of all isolates was assessed by *XbaI*-PFGE. The replicon content was determined by PCR according to the PCR-based replicon typing (PBRT) scheme. Sixty-two non-duplicate ESC-resistant *E. coli* isolates were identified, which all presented an extended-spectrum beta-lactamase (ESBL) phenotype, mostly due to the presence of CTX-M genes. One CTX-M-1-producing *E. coli* was concomitantly colistin-resistant and presented the plasmid-mediated *mcr-1* gene. Nearly all isolates showed associated resistances to non-beta-lactam antibiotics, which could act as co-selectors, even in the absence of beta-lactam use. The results showed a high proportion of ESBL-producing *E. coli* in sheep faeces. Their dissemination was very dynamic, with the spread of successful clones between animals, but also a large diversity of clones and plasmids, sometimes residing in the same animal. This study highlights the need for global surveillance in all food-producing sectors, in order to avoid the dissemination of genes conferring resistance to last-resort antibiotics in human medicine.

## 1. Introduction

The discovery of antibiotics was one of the most important events in the history of human health, but their use and misuse has led to resistances to all known classes of molecules; antimicrobial resistance (AMR) has become a risk to global health [1,2]. The issue of AMR falls into the One Health concept, which postulates that the human, veterinary, and environmental sectors are interconnected [3,4,5]. Resistance to extended-spectrum cephalosporins (ESC) and colistin is of specific concern since these molecules, which are considered critically important antibiotics to human health, have now disseminated into both animals and the environment.

The localisation of resistance genes on plasmids largely favors their ability to be readily transmitted in an inter- or intra-species manner by horizontal transfer [6,7,8,9,10,11,12]. This is very often the case for extended-spectrum beta-lactamase (ESBL) determinants (mostly *bla*_CTX-M_ genes) and the plasmid-borne colistin-resistance genes belonging to the *mcr* gene family. For example, the *mcr-1* gene has been nearly exclusively found on plasmids from the incompatibility (Inc) groups IncX4, IncI2, and IncHI2 [13]. In contrast, *bla*_CTX-M_ genes have been detected on a much larger variety of plasmids, IncF and IncI1 being among the most frequently identified ones [13].

Food-producing animals are an important reservoir of AMR, having a high impact on the whole ecosystem due to their place at the human–animal–environment interface. AMR can disseminate through the food chain, habitat sharing or the spreading of farm waste as landfill [6,14,15]. Sheep production, with an estimated 1 billion animals reared worldwide, is highly important in the livestock field because production is intended for both the food and textile industry [16]. The problem of AMR affects sheep production, just as it does all other farming systems. However, even though a few studies reported resistant bacteria in sheep, including in Portugal [17,18,19,20,21,22,23,24,25], this sector has been much less investigated than bovine, pig, or poultry farming.

In this context, and with a One Health perspective, our study aimed firstly at evaluating the presence of ESC- and colistin-resistant *E. coli* in healthy sheep from Portugal, and secondly at characterizing the collected isolates.

## 2. Results

### 2.1. ESBL-Producing Escherichia coli

#### 2.1.1. Selection and Resistance Profile

**Figure 1:**
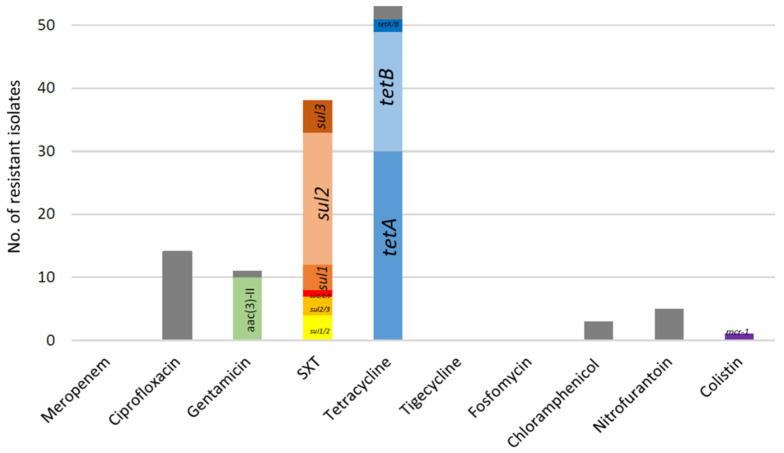
Resistances associated to the 62 ESBL-producing *E. coli* from sheep from Portugal. In grey are resistance phenotypes for which the responsible gene was not looked for or not identified by PCR. Genes conferring low-level resistance to ciprofloxacin (*qnr* gene family and *aac(6′)Ib-cr*) were not included here since chromosomal mutations in the *gyrA* and *parC* genes were not looked for. SXT: sulfamethoxazole-trimethoprim.

ESC-resistant *E. coli* were identified in 19/21 animals (90.5%) on this farm. One to nine non-duplicate ESC-resistant bacteria were found per faecal sample; 62 isolates were obtained in total (Supplementary Materials Table S1). All identified bacteria presented the typical synergy of extended-spectrum-beta-lactamase (ESBL) production and were multi-drug resistant (MDR) according to the definition by Magiorakos et al. [26], i.e., resistance to three or more antibiotic categories, where beta-lactams are divided into six different categories. Only three isolates presented no resistance to non-beta-lactam antibiotics, while 11 isolates were resistant to only one non-beta-lactam antibiotic (tetracycline, *n* = 8; sulfamethoxazole-trimethoprim, *n* = 3). The most frequent associated resistances were to tetracycline (53/62, 85.5%) and sulfamethoxazole-trimethoprim (61.3%), followed by resistances to ciprofloxacin (27.4%), gentamicin (17.7%), nitrofurantoin (8.1%), and chloramphenicol (4.8%) (Figure 1). No resistance was observed for meropenem, tigecycline, and fosfomycin.

#### 2.1.2. Beta-Lactamases and Accessory Resistance Genes

The ESBL phenotype was largely due to the presence of *bla*_CTX-M_ genes (93.5%), and only five *bla*_SHV-12_ genes were identified (including one concomitantly found with a *bla*_CTX-M-32_ gene). Among *bla*_CTX-M_ genes, five variants were identified (Table 1), with a predominance of *bla*_CTX-M-32_ (41.9%) and *bla*_CTX-M-15_ (40.3%). A diversity of other resistance genes was detected (Figure 1 and Table S1), such as *aac(6′)Ib-cr* (35.5%), *qnrS* (3.2%), *aac(3′)-II* (16.1%), *aph(3′)-III* (4.8%), *tetA* (51.6%), *tetB* (33.9%), *sul1* (19.3%), *sul2* (43.5%), and *sul3* (16.1%). The identified genes were coherent with the observed phenotypes.

#### 2.1.3. Plasmid Content

Sixteen replicon types were present in the 62 ESBL-producing isolates, namely FII (*n* = 32) (including 3 FIA and 32 FIB), I1γ (*n* = 27), I1α (*n* = 13), HI1 (*n* = 3), X1 (*n* = 2), X2 (*n* = 19), X3 (*n* = 1), K (*n* = 2), L (*n* = 35), Y (*n* = 23), P (*n* = 2), B/O (*n* = 1), N (*n* = 8), and R (*n* = 1).

The 25 CTX-M-15-producing *E. coli* frequently carried the IncFIB (*n* = 21), IncY (*n* = 19), IncL (*n* = 19), IncFII (*n* = 18), and IncX2 (*n* = 17) replicon types, while the 26 CTX-M-32 producers (including the CTX-M-32 + SHV-12 producer) carried IncL (*n* = 15), IncI1γ (*n* = 13), IncI1α (*n* = 7), and IncFII (*n* = 7). The CTX-M-1- and SHV-12 producers mostly carried the IncI1γ replicon, which was present in three and five isolates, respectively.

#### 2.1.4. Typing and Clonal Relation of ESBL-Producing *E. coli*

The phylogenetic analysis showed the absence of phylogroup B2 and the predominance of group A (*n* = 46), followed by B1 (*n* = 15) and D (*n* = 1). A high number of pulsed-field gel electrophoresis (PFGE) patterns (*n* = 33) was observed (Table 1 and Supplementary Materials Figure S1), highlighting a non-clonal population in this farm. Most PFGE profiles were singletons, but seven clusters (named F, H, L, S, AC, AE, and AF) encompassed clonal isolates identified in 2 to 14 different animals.

### 2.2. Detection of One mcr-1-Positive E. coli

The presence of the *mcr-1* gene in the CTX-M-1-producing *E. coli* Ov11 was confirmed by Sanger sequencing. This isolate presented a colistin minimal inhibitory concentration (MIC) of 4 mg/mL, in coherence with the presence of the *mcr-1* gene. In addition to colistin- and ESC resistance, Ov11 presented resistance to nitrofurantoin and an intermediate profile to ciprofloxacin and gentamicin. This intermediate profile can be explained by the presence of *aac(6′)Ib-cr* gene. Ov11 belonged to the phylogroup B1, had a unique PFGE pattern, and carried the IncI1γ and IncX1 plasmids.

## 3. Discussion

ESBL-producing *Enterobacterales* are a serious threat to human health according to the Centers for Disease Control and Prevention (CDC), with high impact on hospitalisations and healthcare costs [27]. ESBL-producing *E. coli* broke the barriers of the healthcare settings a long time ago and are now present in all compartments of the ecosystem, so that they are often sought as a marker of AMR burden [6,11,28]. While ESBL-producing *E. coli* have been recurrently reported in the poultry, pig, and bovine sectors, sheep farming has been under much less scrutiny. ESBL-producing *E. coli* have been reported in sheep in Chile, Pakistan, Switzerland, Tanzania, Turkey, and the United Kingdom [20,21,22,23,24,25], with proportions ranging from 1.5% to 11.1%. The presence of CTX-M-1- and CTX-M-32-producing *E. coli* in sheep was reported once from the centre of Portugal [19].

Here, we report the massive colonisation (90.5%) of sheep in one Portuguese farm. This is a surprisingly high proportion since animals were reared in an extensive farming system, being led to pastures each day. It was unfortunately not possible to trace the potential use of antibiotics. Of note, all but one *E. coli* belonged to the phylogroup A and B1, which are considered as commensals. Most of the animals presented more than one ESBL-producing *E. coli* strain, with up to nine different isolates in the same sheep. In certain cases, the presence of the same ESBL gene suggested its spread in the *E. coli* gut population of the sheep through horizontal gene transfer. However, different ESBL genes were also identified, proving that one animal can serve as a host for several ESC-resistance genes. PFGE analysis showed the dissemination of a few successful clones between animals, as particularly exemplified by the occurrence of the same PFGE pattern in 14 different animals. In parallel, a large diversity of ESBL-producing *E. coli* was also evidenced, suggesting plasmidic transmission among different genetic backgrounds. All these parameters undoubtedly contributed to the epidemiological success of ESBLs in that farm.

Fifty-nine (95.2%) isolates also displayed resistance phenotypes to non-beta-lactam antibiotics, so ESC resistance can be vertically and/or horizontally selected by the use of other classes of antibiotics. This is especially true for antibiotics such as tetracycline and sulfamethoxazole-trimethoprim, which are largely used in veterinary medicine, and which are also the ones for which we have identified here the greatest number of resistance phenotypes. The limitation of our study lies in the fact that only one farm was sampled, and our results are thus show the urgent need for a tighter survey in this sector.

As with the single report in Portugal, we identified *bla*_CTX-M-1_ and *bla*_CTX-M-32_ in sheep, but we also identified the *bla*_CTX-M-15_, *bla*_CTX-M-14_, *bla*_CTX-M-98_, and *bla*_SHV-12_ genes. CTX-M-15 is the most prevalent ESBL worldwide and particularly in humans in Europe [11,29,30]. Nevertheless, the presence of CTX-M-15 in food-producing animals is no longer an exception, even though CTX-M-1 is still more prevalent in animals [28]. In sheep, CTX-M-15 has been reported only in Switzerland, Tanzania, Turkey, and the United Kingdom [20,21,22,24]. The CTX-M-32 enzyme has emerged in the last years in the food-producing sector [19,28,31,32], and our study thus confirms its importance in livestock.

Among the 25 CTX-M-15-producing *E. coli* isolates in our study, 22 harboured IncF plasmids. IncF plasmids are known to be very successful vectors of the *bla*_CTX-M-15_ genes (an important example is their broad occurrence in ST131 clones) and have disseminated worldwide in the human, animal, and environmental sectors [13,33]. On the contrary, the *bla*_CTX-M-32_ genes were often found on IncN plasmids, which were identified in only 5/26 of our isolates [34,35]. However, in this study, the characterisation of plasmids remains to be performed and only poor information on the ESBL-carrying plasmid can be inferred from the replicon content.

The plasmid-mediated colistin-resistance gene *mcr-1* has first been reported in food-producing animals worldwide [10,36]. Colistin is considered a last-resort option for human infections caused by carbapenem-resistant Gram-negative microorganisms, and is, for this reason, classified by the World Health Organisation (WHO), together with other antibiotics such as last-generation cephalosporins and fluoroquinolones, as a critically important antibiotic for human medicine [12,37]. Fortunately, *mcr-1* genes are still rare in both hospital settings and the community worldwide, thus reinforcing the importance of their surveillance to avoid any emergence in humans. The *mcr-1* gene has been described from various livestock sources (cattle, pigs, poultry, fish, milk, cheese, and eggs) as well as from pets and wild animals, [7,9,36,38,39,40,41,42,43], but our study is its first description in sheep. The Ov11 strain did not contain the main replicons (IncX4, IncHI2, and IncI2) usually described as carrying the *mcr-1* gene, so a chromosomal location cannot be excluded. The surveillance and monitoring of *mcr-1* remains essential in a One Health approach of AMR, to avoid its spread in a sector that presents high proportions of ESBL-producing *E. coli* but still very few resistances to colistin [14].

The importance of food-producing animals in AMR spread cannot be overlooked, since their role as a reservoir has been recurrently demonstrated [5,28]. In line with the results presented here, it is essential and urgent to implement holistic approaches to understand the drivers, dynamics, and epidemiology of AMR in all food-producing systems [4,44].

## 4. Materials and Methods

### 4.1. Sampling Scheme

In 2016, 21 faecal samples were collected from one farm in southern Portugal. Animals were reared in an extensive farming system and were led each day to the pastures. Fresh animal faeces were collected in sterile containers and transported at 4 °C to the laboratory, where they were stored at −20 °C for further characterisation.

### 4.2. Bacterial Isolation and Identification

Samples were thawed and 2 g of each sample of faeces was inoculated into 40 mL of either Tryptic Soy Broth (TSB, Liofilchem, Roseto degli Abruzzi, Italy) or TSB containing a cefotaxime 30 µg disk (Liofilchem, Roseto degli Abruzzi, Italy) and incubated for 24 h at 37 °C. A volume of 100 µL of each enrichment step was plated on MacConkey Agar (MAC, Liofilchem, Roseto degli Abruzzi, Italy) supplemented with cefotaxime (2 µg/mL). One colony per morphology was picked from selective media for further analysis. Samples were plated on antibiotic-free MAC as a growth control. One colony per morphology was picked and plated onto CHROMagar Orientation (CHROMagar, Paris, France) for presumptive identification, and identification was further confirmed by API 20E galleries (Biomérieux, Marcy l’Etoile, France). All isolates were stored in TSB + 20% glycerol for further characterisation.

### 4.3. Antimicrobial Susceptibility Testing and Identification

A total of 18 antibiotics were used in the susceptibility test, by disk diffusion, with amoxicillin, amoxicillin and clavulanic acid, cefoxitin, cefotaxime, ceftazidime, ceftiofur, cefepime, ceftaroline, aztreonam, meropenem, ciprofloxacin, gentamicin, sulfamethoxazole-trimethoprim, tigecycline, tetracycline, fosfomycin, chloramphenicol, and nitrofurantoin (Liofilchem, Roseto degli Abruzzi, Italy). The interpretation was performed according to CLSI [45]. Tigecycline resistance was interpreted according to EUCAST [46]. *E. coli* ATCC 25922 was used as quality control. In accordance with EUCAST standards, the minimal inhibitory concentration (MIC) for colistin was determined by the broth microdilution method [46]. MDR profiles were determined according to standard criteria [26]. ESBL detection was performed using the double-disk synergy test (DDST) [45].

### 4.4. Antimicrobial Resistance Genes

The genes *mcr-1* to *-5* were screened by PCR for all resistant isolates obtained [47]. ESBL-producing isolates were screened for the presence of *bla*_TEM_, *bla*_SHV_, *bla*_OXA_, and *bla*_CTX-M_ (groups 1, 2, 8, 9, and 25) by PCR [48,49]. Isolates with positive amplifications were subjected to Sanger sequencing (Genewiz, Bishop’s Stortford, UK). PCR assays were performed using Super Hot Master Mix (Bioron, Römerberg, Germany); the primers used are listed in Supplementary Materials Table S2.

PCR assays were also performed to determine the presence of other accessory resistance genes: *qnrA*, *qnrB*, *qnrS*, *aac(6′)-Ib-cr*, *aac(3)-II*, *aac(3)-IV*, *ant(2″)-I*, *aph(3′)-I*, *aph(3′)-II*, *aph(3′)-III*, *sul1*, *sul2*, *sul3*, *tetA*, and *tetB* (Table S2).

### 4.5. E. coli Characterisation and Plasmid Content

Phylogenetic groups were detected by PCR as previously described [50]. Pulsed-field gel electrophoresis (PFGE) was performed on all ESBL-producing *E. coli* isolates using the restriction enzyme *XbaI* (Bioron, Römerberg, Germany) [51]. DNA fingerprints were analyzed and the dendrogram of patterns was made using the Dice correlation coefficient, with tolerance and optimisation set at 0.5% and 1%, respectively (BioNumerics, Sint-Martens-Latem, Belgium). Replicon types were detected using the PBRT 2.0 kit (Diatheva, Fano, Italy).

## 5. Conclusions

We showed the massive colonisation of sheep by ESBL-producing *E. coli* in one farm in the south of Portugal. Clonal dissemination was evidenced, as well as the circulation of a wide variety of ESBL-producing *E. coli* clones, highlighting a very dynamic situation. The *mcr-1* gene was also identified in this farm, and for the first time in sheep. Our results proved once more the essential role of monitoring AMR dissemination in all sectors of the One Health continuum, in order to avoid the selection and emergence of resistant pathogens, which is of strategic importance for human health.

## Figures and Tables

**Table 1:** Characteristics of all ESBL-producing isolates collected from sheep in Portugal.

	ESBL Enzyme (*n* = 62)		Phylogroup (*n* = 62) ^1^			No. of Animals (*n* = 21)	No. of PFGE Patterns
	*n*	%	A (*n* = 46)	B1 (*n* = 15)	D (*n* = 1)		
CTX-M-32	25	40.3	19	5	1	15	15
CTX-M-15	25	40.3	23	2	0	16	11
CTX-M-1	5	8.1	1	4	0	4	4
CTX-M-14	1	1.6	1	0	0	1	1
CTX-M-98	1	1.6	1	0	0	1	1
CTX-M-32 + SHV-12	1	1.6	0	1	0	1	1
SHV-12	4	6.5	1	3	0	4	3

^1^ The phylogroup B2 was not included since it was not detected in our collection.

## Supplementary Materials

The following are available online at https://www.mdpi.com/article/10.3390/antibiotics10111403/s1, Figure S1: Clonal relation of 62 ESBL-producing *E. coli* from sheep from Portugal. Table S1: Characterization of 62 ESBL-producing *E. coli* from sheep from Portugal. Table S2: List of primers used in the standard PCRs assays. References [52,53,54,55,56,57,58,59,60,61,62,63,64,65] are cited in the supplementary materials.
