# Supplementary material for: First Global Report of Plasmid-Mediated mcr-1 and Extended-Spectrum Beta-Lactamase-Producing Escherichia coli from Sheep in Portugal
Source: Antibiotics (Basel). 2021 Nov 16;10(11):1403. doi: 10.3390/antibiotics10111403 (PMC8615169; doi:10.3390/antibiotics10111403)
Supplement: Supplementary file 1 [file antibiotics-10-01403-s001.zip › antibiotics-1451679-supplementary.pdf]

Supplementary Material

Table S1. Characterization of 62 ESBL-producing *E. coli* from sheep from Portugal

Animal	Isolate	ESBL enzyme	β-lactam resistance	Other resistance	Other resistance genes	Replicon content (Inc)	PG	PFGE pattern
PTOV01	Ov7	SHV-12	AML, CTX, CAZ, EFT, CPT, ATM	TET	*tetA*	B/O, FIB, I1γ, K, FII	B1	L
PTOV02	Ov2	CTX-M-1	AML, CTX, EFT, FEP, CPT	-	-	L	A	A
PTOV02	Ov3	CTX-M-32	AML, CTX, EFT, CPT	GEN, TET	*aac(6')Ib-cr, aac(3')-II, aph(3')-III, tetA, tetB*	HI1, FII	A	AE
PTOV02	Ov25	CTX-M-15	AML, AMC, CTX, EFT, CPT, ATM	SXT, TET	*sul2, tetA*	FIB, Y, X2, FII	A	AC
PTOV03	Ov4	CTX-M-98	AML, CTX, EFT, CPT	CIP, SXT, TET	*aac(6')Ib-cr, sul1, sul2, tetA*	FIB, FIA, I1γ, FII	A	M
PTOV03	Ov5	CTX-M-15	AML, AMC, CTX, EFT, CPT, ATM	SXT, TET	*sul2, tetA*	FIB, L, Y, X2, FII	A	AC
PTOV03	Ov6	CTX-M-32	AML, AMC, CTX, EFT, CPT	SXT, TET	*sul3, tetB*	FIB, L, X2, FII	A	H
PTOV04	Ov8	CTX-M-15	AML, AMC, CTX, EFT, FEP, CPT, ATM	SXT, TET	*sul2, tetA*	FIB, L, Y, X2, FII	A	AC
PTOV04	Ov9	CTX-M-15	AML, AMC, CTX, CAZ, EFT, CPT, ATM	SXT, TET	*sul2, tetA*	FIB, L, Y, X2, FII	A	AC
PTOV04	Ov10	CTX-M-15	AML, AMC, CTX, EFT, CPT, ATM	CIP, SXT, TET	*aac(6')Ib-cr, sul1, tetB*	FIB, FIA, I1γ, Y, FII	A	Q
PTOV05	Ov11	CTX-M-1	AML, AMC, CTX, CAZ, EFT, FEP, CPT, ATM, FOX	FUR, COL	*aac(6')Ib-cr, mcr-1*	I1γ, X1	B1	W
PTOV05	Ov12	CTX-M-15	AML, AMC, CTX, CAZ, EFT, CPT, ATM	SXT, TET	*sul2, tetA*	FIB, L, X2	A	Z
PTOV05	Ov13	CTX-M-32	AML, CTX, CAZ, EFT, FEP, CPT, ATM	CIP, GEN, TET, FUR	*aac(6')Ib-cr, aac(3)-II, sul1, tetB*	L	A	AF
PTOV05	Ov64	CTX-M-32	AML, AMC, CTX, CAZ, EFT, FEP, CPT, ATM	CIP, GEN, TET	*aac(6')Ib-cr, aac(3)-II, tetB*	I1α, L, I1γ	A	S
PTOV06	Ov14	CTX-M-14	AML, AMC, CTX, EFT, CPT	CIP, SXT, TET	*aac(6')Ib-cr, sul1, sul2, tetA*	I1α, FIB, P, I1γ, FII	A	N
PTOV06	Ov15	CTX-M-15	AML, AMC, CTX, CAZ, EFT, CPT, ATM	SXT, TET	*aac(6')Ib-cr, sul2, tetA*	FIB, L, Y, X2, FII	A	AC
PTOV06	Ov16	CTX-M-32	AML, CTX, EFT, CPT, ATM	SXT, TET	*sul1, sul3, tetB*	L	A	AF
PTOV06	Ov65	CTX-M-32	AML, CTX, EFT, CPT, ATM	CIP, GEN, TET	*aac(6')Ib-cr, aac(3)-II, tetB*	I1α, L, I1γ	A	S
PTOV07	Ov17	CTX-M-15	AML, AMC, CTX, EFT, CPT, ATM	SXT, TET	*sul2, tetA*	FIB, L, Y, X2, FII	A	AC
PTOV07	Ov18	CTX-M-32	AML, CTX, EFT, FEP, CPT, ATM	CIP, SXT, TET	*aac(6')Ib-cr, sul3, tetA*	-	B1	X
PTOV07	Ov19	CTX-M-32,	AML, CTX, CAZ, EFT, CPT, ATM	CIP, GEN, TET	*aac(6')Ib-cr, aac(3)-II, tetB*	I1α, L, I1γ	A	D
PTOV07	Ov20	CTX-M-1	AML, AMC, CTX, EFT, CPT,	SXT	*sul2*	I1α, FIB, X1, FII	B1	G
PTOV07	Ov21	CTX-M-15	AML, AMC, CTX, EFT, CPT,	CIP, SXT, TET	*aac(6')Ib-cr, sul3, tetA*	L, X3, FII	A	AG
PTOV07	Ov22	CTX-M-15	AML, AMC, CTX, EFT, CPT,	SXT, TET	*sul2, tetA*	FIB, L, Y, X2, FII	A	AC
PTOV07	Ov23	CTX-M-15	AML, CTX, EFT, CPT	CIP, SXT	*aac(6')Ib-cr, sul2*	I1α, FIB, P, I1γ, FII	A	O
PTOV07	Ov24	CTX-M-15	AML, AMC, CTX, EFT, FEP, CPT, ATM	SXT, TET	*sul2, tetA*	FIB, L	A	AA
PTOV07	Ov66	CTX-M-32	AML, AMC, CTX, EFT, CPT	GEN, TET	*aac(6')Ib-cr, aac(3)-II, aph(3)-III, tetA, tetB*	HI1, FII	A	AE
PTOV08	Ov26	CTX-M-32	AML, CTX, EFT, FEP, CPT, ATM	TET	*tetA*	N, FIB, I1γ, FII	B1	C
PTOV08	Ov27	CTX-M-15	AML, CTX, CAZ, EFT, CPT, ATM	CIP, SXT, TET	*aac(6')Ib-cr, sul1, tetB*	FIB, FIA, I1γ	A	R
PTOV08	Ov28	CTX-M-15	AML, AMC, CTX, EFT, CPT, ATM	TET	*tetA*	FIB, L, Y, X2, FII	A	AC
PTOV08	Ov29	CTX-M-32	AML, AMC, CTX, CAZ, EFT, CPT, ATM	TET	*tetB*	N, I1γ, FII	A	U
PTOV10	Ov30	CTX-M-32	AML, AMC, CTX, EFT, CPT	SXT, TET, FUR	*sul1, tetB*	L	A	AF
PTOV11	Ov31	CTX-M-32	AML, AMC, CTX, EFT, CPT, ATM	SXT, TET, CHL	*sul1, sul2*	L	A	AF
PTOV11	Ov32	CTX-M-32	AML, AMC, CTX, CAZ, EFT, FEP, CPT, ATM	-	-	N, I1γ	D	AB
PTOV11	Ov33	CTX-M-15	AML, AMC, CTX, CAZ, EFT, FEP, CPT, ATM	SXT, TET	*aac(6')Ib-cr, sul2, tetA*	FIB, L, Y, X2, FII	A	AC
PTOV12	Ov34	CTX-M-1	AML, CTX, EFT, CPT, ATM	TET	*tetB*	N, I1γ	B1	P
PTOV13	Ov35	CTX-M-32	AML, AMC, CTX, EFT, FEP, CPT, ATM	CIP, SXT, TET	*sul3*	R	B1	T

PTOV13	Ov36	CTX-M-32	AML, AMC, CTX, CAZ, EFT, CPT, ATM	-	-	N, L	A	I
PTOV13	Ov37	SHV-12	AML, CTX, CAZ, ATM	TET	*tetA*	N, FIB, I1γ, K, FII	B1	L
PTOV13	Ov38	SHV-12	AML, CTX, CAZ, ATM	CIP, TET	-	I1α, N, FIB, I1γ, Y, X2, FII	A	V
PTOV13	Ov39	CTX-M-15	AML, AMC, CTX, EFT, FEP, CPT, ATM	SXT	*sul2*	FIB, L, Y, X2, FII	A	AC
PTOV13	Ov40	CTX-M-15	AML, CTX, CAZ, EFT, FEP, CPT, ATM	SXT, TET	*sul2, tetA*	I1γ, Y	B1	F
PTOV15	Ov67	CTX-M-32	AML, AMC, CTX, CAZ, EFT, CPT, ATM	CIP, GEN, TET	*aac(6')Ib-cr, aac(3)-II, tetB*	I1α, L, I1γ	A	S
PTOV15	Ov42	CTX-M-15	AML, AMC, CTX, CAZ, EFT, FEP, CPT, ATM	GEN, SXT, TET, FUR	*aac(6')Ib-cr, qnrS, aph(3)-III, sul2, tetA*	FIB, L, Y, X2, FII	A	AC
PTOV15	Ov44	CTX-M-32	AML, AMC, CTX, CAZ, EFT, CPT, ATM	GEN	*aac(6')Ib-cr, aac(3)-II*	I1α, I1γ	B1	E
PTOV15	Ov45	CTX-M-32, SHV-12	AML, AMC, CTX, CAZ, ATM	SXT, TET	*sul1, sul2, tetA*	I1γ, Y	B1	J
PTOV16	Ov46	CTX-M-1	AML, AMC, CTX, EFT, CPT,	TET	*tetA, tetB*	I1α, FIB, I1γ, FII	B1	K
PTOV16	Ov47	CTX-M-15	AML, AMC, CTX, CAZ, EFT, CPT, ATM	SXT, TET	*sul2, tetA*	FIB, L, Y, X2, FII	A	AC
PTOV17	Ov49	CTX-M-32	AML, AMC, CTX, EFT, FEP, CPT, ATM	SXT, TET	*sul3, tetB*	FIB, Y, FII	A	H
PTOV17	Ov51	CTX-M-15	AML, CTX, CAZ, EFT, CPT, ATM	SXT	*sul2, sul3*	FIB, L, Y, X2, FII	A	AC
PTOV17	Ov52	SHV-12	AML, CTX, CAZ, EFT, ATM	TET, CHL	*tetA*	I1α, I1γ	B1	Y
PTOV17	Ov63	CTX-M-32	AML, AMC, CTX, CAZ, EFT, CPT, ATM	CIP, GEN, TET	*aac(6')Ib-cr, aac(3)-II, tetB*	I1α, L, I1γ	A	S
PTOV18	Ov53	CTX-M-15	AML, CTX, EFT, CPT	TET	*tetA*	L	A	AD
PTOV18	Ov54	CTX-M-15	AML, AMC, CTX, EFT, CPT	SXT, TET	*sul2, tetA*	FIB, L, Y, X2	A	AC
PTOV18	Ov55	CTX-M-15	AML, CTX, EFT, CPT, ATM	SXT, TET	*sul2, tetA*	I1γ, Y	B1	F
PTOV18	Ov56	CTX-M-32	AML, CTX, EFT, CPT, ATM	SXT, TET	*sul1, sul3, tetB*	L	A	AF
PTOV19	Ov57	CTX-M-32	AML, CTX, EFT, CPT	SXT, TET	*sul1, sul3, tetB*	L	A	AF
PTOV19	Ov58	CTX-M-32	AML, CTX, CAZ, EFT, FEP, CPT, ATM	SXT, TET, CHL	*sul1, sul2, sul3, tetB*	HI1, FIB, I1γ, FII	A	B
PTOV19	Ov59	CTX-M-15	AML, CTX, CAZ, EFT, FEP, CPT, ATM	SXT, TET	*sul2, tetA*	FIB, L, Y, X2, FII	A	AC
PTOV20	Ov61	CTX-M-15	AML, AMC, CTX, CAZ, EFT, FEP, CPT, ATM	CIP, SXT, TET, FUR	*aac(6')Ib-cr, qnrS, sul2, tetA*	FIB, L, Y, X2, FII	A	AC
PTOV20	Ov69	CTX-M-32	AML, AMC, CTX, CAZ, EFT, CPT, ATM	CIP, GEN, TET	*aac(6')Ib-cr, aac(3)-II, tetB*	I1α, L, I1γ	A	S
PTOV21	Ov62	CTX-M-32	AML, CTX, CAZ, EFT, FEP, CPT, ATM	SXT, TET	*sul2, tetA*	N, L, I1γ, Y	B1	F

AML – amoxicillin, AMC – amoxicillin-clavulanic acid, CTX – cefotaxime, CAZ – ceftazidime, EFT – ceftiofur, FEP – cefepime, CPT – ceftaroline, ATM – aztreonam, FOX – cefoxitin, CIP – ciprofloxacin, GEN – gentamicin, SXT – sulfamethoxazole-trimethoprim, TET – tetracycline, CHL – chloramphenicol, FUR – nitrofurantoin.
PG: phylogroup.

Table S2: List of primers used in the standard PCRs assays.

Aim	Target	Primer Name	Primer Sequence (5′-3′)	Amplicon Size (pb)	*Annealing* (T)	Reference
Colistin resistance	*mcr-1*	MCR-1 F	AGTCCGTTTGTTCTTGTGGC	320	58°C	[47]
		MCR-1 R	AGATCCTTGGTCTCGGCTTG			
	mcr-2	MCR2-F	CAAGTGTGTTGGTCGCAGTT	715	58°C	[47]
		MCR2-R	TCTAGCCCGACAAGCATACC			
	mcr-3	MCR3-F	AAATAAAAATTGTTCCGCTTATG	929	58°C	[47]
		MCR3-R	AATGGAGATCCCCGTTTTT			
	mcr-4	MCR4-F	TCACTTTCATCACTGCGTTG	1116	58°C	[47]
		MCR4-R	TTGGTCCATGACTACCAATG			
	mcr-5	MCR5-F	ATGCGGTTGTCTGCATTTATC	1644	58°C	[47]
		MCR5-R	TCATTGTGGTTGTCCTTTTCTG			
Beta-lactamases	bla_{CTX-M}G1	MP CTX-M G1 F	AAA AAT CAC TGC GCC AGT TC	415	52°C	[48]
		MP CTX-M G1 R	AGC TTA TTC ATC GCC ACG TT			
	bla_{CTX-M}G2	MP CTX-M G2 F	CGA CGC TAC CCC TGC TAT T	552	52°C	[48]
		MP CTX-M G2 R	CCA GCG TCA GAT TTT TCA GG			
	bla_{CTX-M}G8	MP CTX-M G8 F	CGT TAA GCG GAT GAT GC	666	52°C	[48]
		MP CTX-M G8 R	AAC CCA CGA TGT GGG TAG C			
	bla_{CTX-M}G9	MP CTX-M G9 F	CAA AGA GAG TGC AAC GGA TG	205	52°C	[48]
		MP CTX-M G9 R	ATT GGA AAG CGT TCA TCA CC			

	$bla_{\text{CTX-M}}$G25	MP CTX-M G25 F	GCA CGA TGA CAT TCG GG	327	52°C	[48]
		MP CTX-M G25 R	AAC CCA CGA TGT GGG TAG C			
	bla_{OXA}	OXA F	CATTTCCGTGTCGCCCTTATTC	800	57°C	[49]
		OXA R	CGTTCATCCATAGTTGCCTGAC			
	bla_{TEM}	TEM F	AGCCGCTTGAGCAAATTAAAC	713	57°C	[49]
		TEM R	ATCCCGCAGATAAATCACCAC			
	bla_{SHV}	SHV F	GGCACCAGATTCAACTTTCAAG	564	57°C	[49]
		SHV R	GACCCCAAGTTTCCTGTAAGTG			
Quinolone resistance	*qnrA*	qnrA F	GGATGCCAGTTTCGAGGA	492	59°C	[52]
		qnrA R	TGCCAGGCACAGATCTTG			
	qnrB (1-6)	qnrB (1-6) F	GGMATHGAAATTCGCCACTG	580	57°C	[53]
		qnrB (1-6) R	TTTGCYGYYCGCCAGTCGAA			
	qnrS (1-2)	qnrS (1-2) R	GATCTAAACCGTCGAGTTCGG	466	57°C	[52]
		qnrS (1-2) F	TCGACGTGCTAACTTGCG			
	aac6'Ib-cr	aac-cr- F	TTGCGATGCTCTATGAGTGG	400	57°C	[54]
		aac-cr-R	GCGTGTTCGCTCGAATGCC			
Aminoglycoside resistance	*aac(3)-II*	aac(3)-II 1	GTCGAACAGGTAGCACTGAG	568	63°C	[55]
		aac(3)-II 2	TGAAACGCTGACGGAGCCTC			
	aac(3)-IV	aac(3)-IV- F	GTGTGCTGCTGGTCCACAGC	705	55°C	[56]
		aac(3)-IV- R	AGTTGACCCAGGGCTGTCGC			
	ant(2'')-I	ant(2'')-I F	GGGCGCGTCATGGAGGAGTT		68°C	[57]
		ant(2'')-I R	TATCGCGACCTGAAAGCGGC			
	aph(3')-I	aph(3')-I F	GCCGATGTGGATTGCGAAAA		68°C	[58]
		aph(3')-I R	GCTTGATCCCCAGTAAGTCA			
	aph(3')-II	aph(3')-II F	GCTATTCGGCTATGACTGGGC		63°C	[59]
		aph(3')-II R	CCACCATGATATTCGGCAAGC			
	aph(3')-III	aph(3')-III F	AACGTCTTGCTCGAGGCCGCG		53°C	[60]
		aph(3')-III R	GGCAAGATCCTGGTATCGGTC			
Tetracycline resistance	*tetA*	tetA 1	GTAATTCTGAGCACTGTCGC	950	57°C	[61]
		tetA 2	CTGCCTGGACAACATTGCTT			
	tetB	tetB 1	CTCAGTATTCCAAGCCTTTG	430	53°C	[62]
		tetB 2	ACTCCCCTGAGCTTGAGGGG			
Sulphonamide resistance	*sul1*	sul1 F	TGAGATCAGACGTATTGCGC	400	59°C	[63]
		sul1 R	TTGAAGGTTCGACAGCACGT			
	sul2	sul 2 F	GCGCTCAAGGCAGATGGCATT	270	70°C	[59]
		sul 2 R	GCGTTTGATACCGGCACCCGT			
	sul3	sul 3 F	CATCTGCAGCTAACCTAGGGCTTTGGA	990	53°C	[64]
		sul 3 R	GAGCAAGATTTTTGGAATCG			
E. coli Phylogenetic Group	*chuA*	ChuA.1b	ATGGTACCGGACGAACCAAC	288	59°C	[65]
		ChuA.2	TGCCGCCAGTACCAAAGACA			
	yjaA	YjaA.1b	CAAACGTGAAGTGTCAGGAG	211	59°C	[65]
		YjaA.2b	AATGCGTTCCTCAACCTGTG			
	arpA	AceK.f	AACGCTATTCGCCAGCTTGC	400	59°C	[65]
		ArpA1.r	TCTCCCCATACCGTACGCTA			
	TspE4C2	TspE4C2.1b	CACTATTCGTAAGGTCATCC	152	59°C	[65]
		TspE4C2.2b	AGTTTATCGCTGCGGGTCGC			

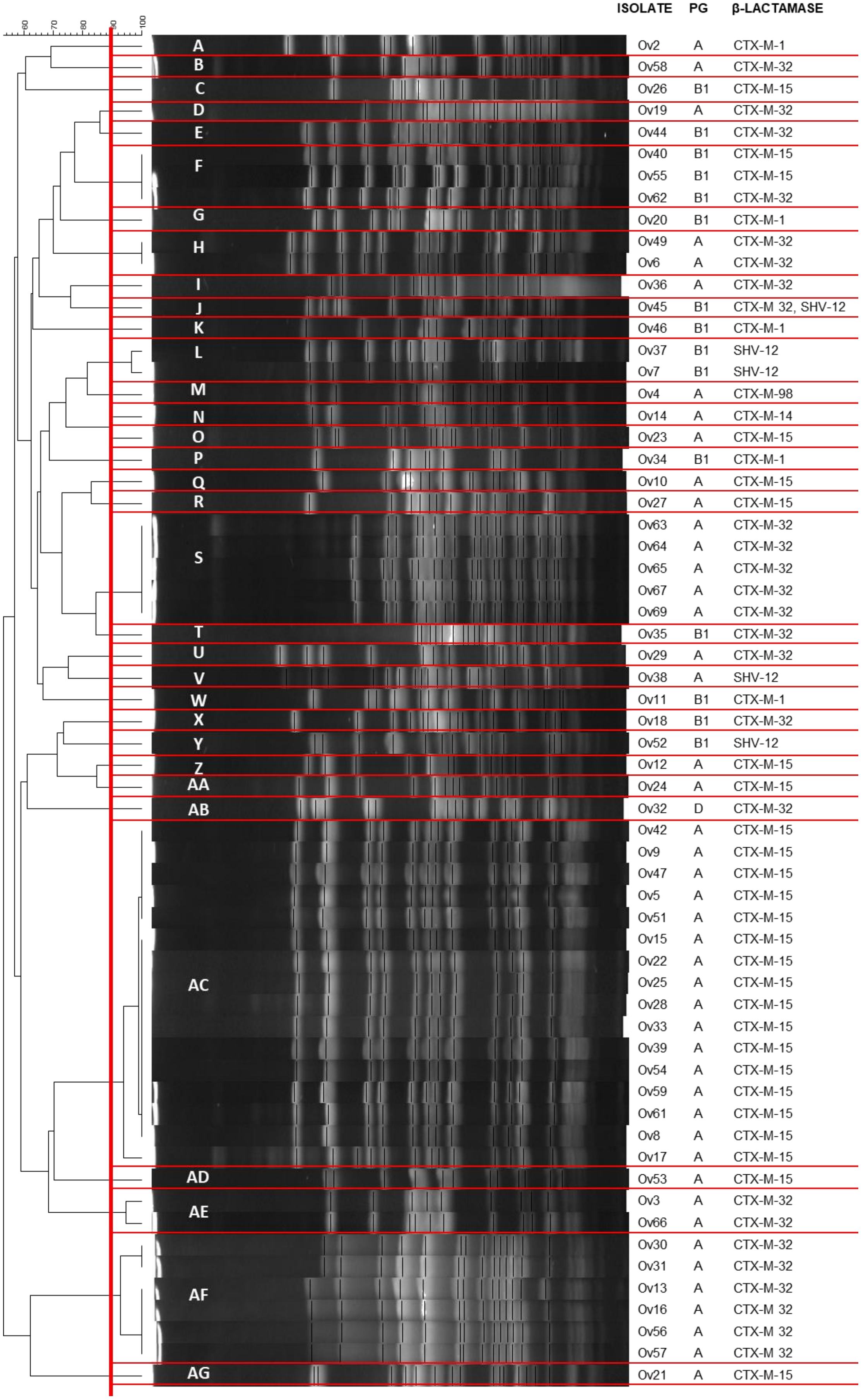

ISOLATE	PG	β-LACTAMASE
Ov2	A	CTX-M-1
Ov58	A	CTX-M-32
Ov26	B1	CTX-M-15
Ov19	A	CTX-M-32
Ov44	B1	CTX-M-32
Ov40	B1	CTX-M-15
Ov55	B1	CTX-M-15
Ov62	B1	CTX-M-32
Ov20	B1	CTX-M-1
Ov49	A	CTX-M-32
Ov6	A	CTX-M-32
Ov36	A	CTX-M-32
Ov45	B1	CTX-M 32, SHV-12
Ov46	B1	CTX-M-1
Ov37	B1	SHV-12
Ov7	B1	SHV-12
Ov4	A	CTX-M-98
Ov14	A	CTX-M-14
Ov23	A	CTX-M-15
Ov34	B1	CTX-M-1
Ov10	A	CTX-M-15
Ov27	A	CTX-M-15
Ov63	A	CTX-M-32
Ov64	A	CTX-M-32
Ov65	A	CTX-M-32
Ov67	A	CTX-M-32
Ov69	A	CTX-M-32
Ov35	B1	CTX-M-32
Ov29	A	CTX-M-32
Ov38	A	SHV-12
Ov11	B1	CTX-M-1
Ov18	B1	CTX-M-32
Ov52	B1	SHV-12
Ov12	A	CTX-M-15
Ov24	A	CTX-M-15
Ov32	D	CTX-M-32
Ov42	A	CTX-M-15
Ov9	A	CTX-M-15
Ov47	A	CTX-M-15
Ov5	A	CTX-M-15
Ov51	A	CTX-M-15
Ov15	A	CTX-M-15
Ov22	A	CTX-M-15
Ov25	A	CTX-M-15
Ov28	A	CTX-M-15
Ov33	A	CTX-M-15
Ov39	A	CTX-M-15
Ov54	A	CTX-M-15
Ov59	A	CTX-M-15
Ov61	A	CTX-M-15
Ov8	A	CTX-M-15
Ov17	A	CTX-M-15
Ov53	A	CTX-M-15
Ov3	A	CTX-M-32
Ov66	A	CTX-M-32
Ov30	A	CTX-M-32
Ov31	A	CTX-M-32
Ov13	A	CTX-M-32
Ov16	A	CTX-M 32
Ov56	A	CTX-M 32
Ov57	A	CTX-M 32
Ov21	A	CTX-M-15

PG – Phylogenetic group

Figure S1. Clonal relation of 62 ESBL-producing *E. coli* from sheep from Portugal
